# Older people’s health literacy on COVID-related health behaviours during the pandemic – Results from the SWEOLD 2021 study in Sweden

**DOI:** 10.1177/14034948251357704

**Published:** 2025-07-16

**Authors:** Naima Kayser Leeoza, Josefin Wångdahl, Carin Lennartsson, Janne Agerholm

**Affiliations:** 1 Aging Research Centre, Karolinska Institutet and Stockholm University, Stockholm, Sweden; 2 Department of Oncology-Pathology, Karolinska Institutet, Stockholm, Sweden; 3 Division of Nursing, Department of Neurobiology, Care Sciences, and Society, Karolinska Institutet, Stockholm, Sweden; 4 Department of Public Health and Caring Sciences, Uppsala University, Uppsala, Sweden

**Keywords:** Health literacy, communicative and critical health literacy, older people, COVID-19, pandemic, health information, recommendations, vaccination, Sweden, SWEOLD

## Abstract

**Background:**

Health literacy refers to an individual’s ability to access, understand, and use information to promote and maintain good health. The importance of a health-literate population has been highlighted during COVID-19 when health authorities pleaded with the public to be responsible and adhere to rules and recommendations. This study investigates the relationship between health literacy levels and older people’s understanding of and adherence to COVID-19 recommendations.

**Methods:**

The data for this study were obtained from the 2021 Swedish Panel Study of Living Conditions of the Oldest Old (SWEOLD), which exclusively includes individuals aged ⩾77 years. Health literacy was assessed utilising the Communicative and Critical Health Literacy (C&CHL) scale. Using logistic regression, we evaluated the association between C&CHL and vaccination coverage, as well as the comprehension of and adherence to health recommendations and the avoidance of healthcare services due to apprehension related to COVID-19.

**Results:**

The findings showed that 90% of the study population understood the COVID-19 guidelines, and 63% adhered to them to a large extent. Further, 98% got vaccinated. Although limited C&CHL was statistically associated with a lower ability to understand information (odds ratio: 2.20), it was not related to following recommendations, being vaccinated, or refraining from health or dental care.

**Conclusions:**

**Regardless of individual health literacy level, most older people understood and followed COVID-19 recommendations, and nearly everyone was vaccinated. The findings indicate that the information about Swedish COVID-19 recommendations and vaccination information was tailored to meet the needs of different health literacy levels.**

## Background

### COVID-19 from a global and Swedish perspective

Like the populations of many other countries, Swedish society was significantly impacted by the novel coronavirus, SARS-CoV-2. The first wave of COVID-19 (between March 2020 and June 2020) infected 19,000 people, and 38% of those cases were people aged 70 and over [1]. Sweden emphasised personal accountability and urged individuals to comply with COVID-19 guidelines, such as maintaining appropriate hygiene and limiting physical and social interactions. Individuals aged 70 and older were explicitly advised to minimise social interactions and adhere to quarantine measures to mitigate the spread [2, 3]. During the spring of 2020, as the pandemic globally escalated at an alarming rate, Sweden implemented moderate measures to control the situation, including imposing restrictions on public gatherings and travel and temporarily shutting down certain enterprises. This approach resulted in a high number of COVID-19-related fatalities, with Sweden recording one of the highest case rates in Europe [2].

In collaboration with various organisations, the Public Health Agency of Sweden disseminated information about COVID-19, its transmission, and preventive measures via public campaigns and standardised templates. These templates included textual and visual support to communicate information and recommendations to the public. They were freely available for download online, could be used by any organisation, and were not restricted to a specific sector. Furthermore, they were printed in multiple languages to maximise their accessibility to a broader audience [3]. This open-access approach enabled wide dissemination and adaptation of the recommendations across diverse settings.

Although not legally binding, these recommendations by the authorities were strongly encouraged. People in Sweden demonstrated a proactive willingness to comply by staying informed about guidelines, practising self-isolation, and adopting preventive measures such as regular handwashing, maintaining a one-metre distance, and covering their nose and mouth when coughing [4]. More information about the step-by-step changes in restrictions during the data collection period can be found elsewhere [3].

### Health literacy and COVID-19

Health literacy (HL) is defined as an individual’s cognitive and social competencies that significantly affect their ability to access, comprehend, evaluate, and utilise information to promote and sustain optimal health [5]. The concept is often divided into three dimensions: functional HL, which refers to the basic skills needed to be able to read and write (e.g., reading health information and filling out medical forms); communicative HL, which refers to more advanced cognitive skills that enable people to interact with healthcare personnel and to actively participate in treatment decisions; and finally critical HL, which refers to the individual’s ability to critically analyse and use information to make choices and to exert greater control over their health and life situation [6].

HL is of the utmost importance in promptly obtaining and following health information and advice during epidemics like COVID-19, and critically evaluating the value of information from multiple sources that may conflict. The substantial influx of knowledge disseminated globally during the COVID-19 pandemic resulted in an infodemic characterised by inaccurate information and unclear communication [7]. Understanding and assessing health information can be complex, resulting in misunderstandings and inappropriate responses to disease prevention.

Limited HL has been linked to poorer adherence to COVID-19 recommendations and preventive measures across multiple countries. It has, for example, been shown that people with limited HL are more likely to find COVID-19 recommendations challenging to understand and are less likely to follow them [8, 9], have negative attitudes towards preventive strategies [10, 11], and are less likely to be aware of symptoms and preventive actions [9]. Furthermore, individuals with limited HL often behave reluctantly towards COVID-19 vaccination [12, 13] and are more afraid of infection [14]. This may be an additional contributing factor to the shift in care-seeking behaviour observed during the COVID-19 pandemic, which has previously been attributed to factors such as low educational attainment, imposed restrictions, and fear of infection [15, 16]. However, while changes in care-seeking may result in forgone care, our focus is specifically on whether individuals *intentionally avoided care due to fear of infection*, which reflects a distinct behavioural response rather than unmet medical needs per se. Further research is needed to determine the role of health literacy in shaping such decisions during public health crises.

While HL is often considered an individual-level skill, effectively supporting populations with varying levels of HL requires a broader societal approach, most notably through improving organisational health literacy (OHL). OHL refers to the extent to which organisations equitably enable individuals to find, understand, and use information and services to inform health-related decisions and actions for themselves and others. Strengthening OHL ensures that recommendations are accessible, relevant, and culturally appropriate [17], which was especially important during the infodemic that accompanied the pandemic [18]. This concept was especially relevant in Sweden during the COVID-19 pandemic, when public authorities, particularly the Public Health Agency (Folkhälsomyndigheten), communicated important health recommendations to the public.

This was not unique to Sweden. As also seen worldwide, specific subpopulations, particularly older people, were disproportionately affected by the COVID-19 pandemic. Research indicates that older age is a significant risk factor for mortality, and the presence of multimorbidity further elevates the risk of severe illness and death from COVID-19 [17, 18]. The older age group often has limited HL and reduced familiarity with modern technology and technical devices [19]. This may have affected this age group more profoundly than younger age groups, given that a substantial portion of COVID-19 information was also communicated online.

No specific studies have been identified in Sweden that address the relationship between health literacy levels and older people’s understanding of and adherence to COVID-19 recommendations. Our research aims to fill this knowledge gap. To prepare for future pandemics, it is crucial to understand how Swedish recommendations were conveyed in an understandable and accessible manner to all older individuals, regardless of their HL level. Therefore, this study aims to investigate the extent to which COVID-19 recommendations were comprehended and followed by older people in Sweden, depending on their communicative and critical health literacy (C&CHL) level. A secondary aim is to explore whether abstaining from health or dental care due to fear of COVID-19 infection is linked to C&CHL levels.

## Methods

### Design and study population

This study is based on data from the Swedish Panel Study of Living Conditions of the Oldest Old (SWEOLD) 2021, a nationally representative survey of individuals aged ⩾77 years. SWEOLD 2021 comprises 848 individuals (response rate: 64%), selected via a combination of follow-up from participants in the Swedish Level of Living Survey (LNU), a nationally representative study, and supplementary random sampling. Supplementary random samples were extracted from the general Swedish population aged 85 and above, who would otherwise have been under-represented, to enhance the statistical power of analyses concerning the oldest age cohorts. Sampling weights were applied to account for different probabilities of inclusion based on age and sex.

Due to the ongoing COVID-19 public health emergency, all participants were invited to participate via telephone interviews conducted by trained interviewers. Among the 848 participants, 203 individuals (24%) were unable to participate independently due to physical or cognitive impairment. For these individuals, a close relative, legal representative, or healthcare provider participated in an indirect interview on their behalf. In these cases, HL questions were not asked.

Therefore, the final analytic sample for this study consisted of 645 participants who responded to the interview themselves: 631 participants (97.8%) completed the interview independently. In comparison, 14 participants (2.2%) received some support from another person but still answered the questions themselves. Data collection took place between May 2021 and April 2022, when many public health recommendations were lifted, although COVID-19 remained classified as a public health emergency in Sweden. Further details on the SWEOLD study are available elsewhere [20].

### Exposure

#### The measure of HL

HL was measured using the C&CHL scale, which had been previously translated into Swedish and validated in a Swedish context [21]. The scale contains five items that assess the participant’s ability to collect and extract information, determine credibility, and make health-related decisions. These items are answered on a five-point Likert scale ranging from strongly agree (=1) to strongly disagree (=5). In line with the manual for the scale [22], those who reported ‘strongly agree’ or ‘agree’ on items were categorised as having sufficient C&CHL; those who answered ‘partially agree’ on all or some of the items were categorised as having problematic C&CHL; and those who answered ‘strongly disagree’ or ‘disagree’ on any item were categorised as having inadequate C&CHL. Thereafter, the variable was dichotomised into sufficient and limited (inadequate and problematic) C&CHL.

### Outcome measures

#### Vaccination for COVID-19

Being vaccinated was assessed with the following question: ‘Have you been vaccinated against COVID-19?’ with the responses ‘Yes, once’, ‘Yes, twice’, ‘No, but I will’, and ‘No, I will not be vaccinated’, which were dichotomised into ‘Yes’ ( by merging ‘Yes once’ and ‘Yes twice’) and ‘No’ (by merging other options).

#### Understanding and following COVID-19 recommendations

Understanding COVID-19-related recommendations was assessed with the following question: ‘If you think about the information you have received regarding recommendations, how easy or hard was it to understand?’ We have dichotomised this by merging the first two responses (‘Very easy’, ‘Easy’) into ‘To a large extent’ and the last two responses (‘Difficult’, ‘Very difficult’) as ‘To a small extent’.

The degree to which people followed COVID-19 recommendations was assessed with the following question: ‘To what degree have you followed the authorities’ recommendations regarding COVID-19?’ with the responses ‘To a large extent’, ‘To a quite large extent’, ‘To a small extent’, and ‘to a quite small extent’, which were dichotomised into ‘To a large extent’ and ‘To a small extent’.

#### Refraining from health or dental care due to fear of COVID-19

Refraining from health or dental care was assessed with the following questions: ‘Have you refrained from seeking medical care in the last 12 months?’ and ‘Have you refrained from seeking dental care in the last 12 months?’ with responses as ‘Yes’ and ‘No’. The questions were followed up with a question about the reason for refraining, where ‘Fear of COVID-19 infection’ was one of the several options, with two responses: ‘Yes’ and ‘No’. This study combined the questions into ‘Refrained from health or dental care due to fear of COVID-19’ (‘Yes’, ‘No’).

### Covariates

Age was measured as a continuous variable and subcategorised into 77–80, 81–85, 86–90, and 91+. Sex was categorised as ‘Male’ and ‘Female’. Educational level was measured as ‘Highly educated’ (beyond grade school) and ‘Lower education’ (grade school or equivalent). Social class was determined using the official Swedish socioeconomic classification (SEI), which is related to the Erikson–Goldthorpe–Portocarero (EGP) classification [23]. SEI is based on the main occupation in the labour market, as reported by the respondent [24]. We categorised social class into five classes: ‘Unskilled workers’, ‘Skilled workers + Lower non-manual workers’, ‘Intermediate non-manual workers’, ‘Self-employed workers and Farmers’, and ‘Higher non-manual workers’.

### Statistical analyses

Descriptive statistics were used to summarise the sociodemographic and COVID-19-related characteristics of the study population. The chi-squared test was used to assess these categorical covariates by the level of C&CHL. Logistic regression models, yielding adjusted odds ratios (AOR) with corresponding 95% confidence intervals (CI), were used to investigate the connections between C&CHL and the outcome variables, particularly understanding and adhering to COVID-19 guidelines, receiving vaccinations, and refraining from health or dental care. The models were adjusted for age, sex, educational level, and social class. We also further analysed whether those who need to follow the recommendation are the ones who require help to understand the information, using crosstabulation between the two variables. All analyses used SPSS version 28.0, with a significance level (α) set at <0.05.

The data was weighted to account for differences in the sample’s and the general population’s age and sex distributions.

### Ethics

Ethical approval was obtained from The Swedish Ethical Review Authority, Karolinska Institutet, Stockholm (Dnr.: 2021-00393).

## Results

Table 1 presents the characteristics of the study population overall and across levels of C&CHL. In our study, 44% of the population was between 80 and 84 years old, with a higher proportion of females (57%) than males. The majority were highly educated (66%) and belonged to either skilled manual workers or lower non-manuals (32%), or intermediate non-manuals (23%). Of our sample, 57% had ‘sufficient C&CHL’, and 44% had ‘limited C&CHL’ levels. Most respondents were vaccinated (98%), understood COVID-19-related instructions (90%), followed COVID-19 instructions (63%), and did not refrain from seeking health or dental care due to the fear of COVID-19 infection (90%). Individuals with low C&CHL levels were more likely to be older, male, and have lower educational and social classes (Table I). People with limited C&CHL were also less likely to find COVID-19-related information easy to understand.

**Table I.** The characteristics of the study population (*n*) and frequency (%) of sociodemographic and COVID-19-related variables by level of C&C HL level (*n*=645).

	Total (*n*)	Weighted total percentage	Sufficient C&CHL (%)	Limited C&CHL (%)	Chi-square test *p*-value
	*n*=645	*n*=493	56.5%	43.5%	
**Sex**					
Man	298	42.90	48.10	37.30	0.02
Woman	347	57.10	51.90	62.70	
**Age (in years)**					
77–79	145	29.40	38.50	21.90	<.001
80–84	216	43.80	43.10	43.30	
85–89	116	18.60	14.50	23.90	
90+	168	8.10	3.80	10.90	
**Educational level** ^a^					
Highly educated	405	66.10	73.80	57.40	<.001
Low educated	220	33.90	26.20	42.60	
**Socioeconomic position**					
Unskilled manual workers	117	19.20	12.10	26.30	<.001
Skilled manual workers and lower non-manual workers	189	31.60	27.20	38.10	
Intermediate non-manual workers	142	23.30	27.60	19.10	
Self-employed workers and farmers	66	8.70	8.90	6.70	
Higher non-manual workers	105	17.30	24.10	9.80	
**Understanding information**					
To a large extent	562	90.40	93.10	86.40	0.017
To a small extent	62	9.60	6.90	13.60	
**Follow recommendation**					
To a large extent	378	62.80	64.60	60.30	0.348
To a small extent	227	37.20	35.40	39.70	
**Vaccinated**					
Yes	628	98.30	98.90	97.50	0.269
No	12	1.70	1.10	2.50	
**Refraining from health or dental care due to fear of COVID-19 infection**					
Yes	59	9.90	8.80	12.90	0.158
No	584	90.10	91.20	87.10	

a Educational level: highly educated (beyond grade school) and low education (grade school or equivalent).

C&CHL: communicative and critical health literacy.

Table II shows the association between C&CHL and the three outcome variables. People with low C&CHL had 2.2 times higher odds of not understanding COVID-19 information than individuals with sufficient C&CHL (OR: 2.20, 95% CI: 1.12–4.32).

**Table II.** Odds ratios (OR) with a 95% confidence interval (CI) for not understanding and following COVID-19 recommendations and refraining from health or dental care due to COVID-19 by level of C&CHL level.

	Not understanding			Not following recommendations			Refraining from health or dental care due to COVID-19		
Variables	Adj. OR	(95% CI)		Adj. OR	(95% CI)		Adj. OR	(95% CI)	
**C&C HL**									
Sufficient	1			1			1		
Limited	**2.20**	**1.12**	**4.32**	0.92	0.60	1.42	1.62	0.85	3.11
**Age**									
77–80 years	1			1			1		
81–85 years	0.81	0.39	1.67	1.47	0.91	2.37	0.83	0.40	1.72
86–90 years	0.41	0.15	1.18	1.43	0.79	2.59	1.16	0.50	2.68
91+	0.57	0.14	2.31	**2.44**	**1.02**	**5.85**	0.19	0.02	1.70
**Sex**									
Male	1			1			1		
Female	0.78	0.41	1.49	**0.64**	**0.43**	**0.97**	1.38	0.72	2.64
**Education level**									
Highly educated	1			1			1		
Low educated	1.13	0.55	2.32	1.12	0.71	1.77	1.04	0.51	2.12
**Socioeconomic position**									
Unskilled manual workers	1			1			1		
Skilled manual workers and lower non-manual workers	0.67	0.28	1.64	0.92	0.52	1.65	0.69	0.28	1.66
Intermediate non-manual workers	1.00	0.37	2.68	**0.44**	**0.22**	**0.87**	0.95	0.36	2.52
Self-employed workers and farmers	1.46	0.46	4.67	0.72	0.31	1.69	1.03	0.29	3.64
Higher non-manual workers	0.29	0.07	1.23	0.62	0.30	1.27	0.78	0.25	2.41
**Understand information**									
To a large extent	–	–	–	1			–	–	–
To a smaller extent	–	–	–	1.35	0.70	2.61	–	–	–

The model was adjusted for age, sex, educational level, and social class. Bold text indicates statistically significant results (*p*-value <0.05).

Not following the COVID-19-related recommendations was statistically significantly associated with age, sex, and socioeconomic position, particularly among intermediate non-manual workers. Females had 0.64 times lower odds than males (95% CI: 0.43–0.97), while those aged 91 and older had 2.44 times greater odds than individuals aged 77–80 (95% CI: 1.02–5.85). Furthermore, intermediate non-manual workers had 0.44 times lower odds than unskilled manual workers (95% CI: 0.22–0.87). Females had 0.64 times lower odds than males (95% CI: 0.43–0.97), and individuals aged 91 and older had 2.44 times higher odds than those aged 77–80 (95% CI: 1.02–5.85). Furthermore, intermediate non-manual workers had 0.44 times lower odds than unskilled manual workers (95% CI: 0.22–0.87).

## Discussion

### Main findings

In this study, we investigated the association between having limited C&CHL and understanding and following COVID-19 recommendations, getting the COVID-19 vaccine, and refraining from health or dental care due to fear of COVID-19.

### Understanding and following COVID-19 recommendations

Our findings showed that in Sweden, most people understood and followed COVID-19 recommendations, which could also indicate that the recommendations provided by the Public Health Agency and other organisations were provided in an HL-friendly way. Limited C&CHL was associated with difficulties understanding COVID-19 recommendations in the adjusted analyses. This aligns with previous studies [9, 11]. However, we found no association between C&CHL and the degree to which people followed the recommendations. Regardless of comprehension, individuals adhered to the recommendation to the same degree. Again, this can be explained by the Swedish authorities’ effective dissemination of information using visual aids and culturally appropriate materials to overcome barriers to understanding health information for groups facing language or literacy challenges. Several articles have backed up this approach, agreeing that integrating culturally driven communication strategies ensures the effective distribution of health messages across diverse demographic groups and fosters better engagement and compliance with public health guidelines [9, 18].

### Getting vaccinated for COVID-19

Almost all participants in this study received a COVID-19 vaccination (98%), which aligns with previous studies on vaccination coverage in older populations [25]. We found no association between C&CHL level and the avoidance of vaccinations. This contrasts with earlier studies on vaccine hesitancy in older people from other countries, revealing that the likelihood of not being vaccinated is linked to limited HL [12, 13]. Our findings suggest that the authorities have effectively communicated the significance of vaccination to older people and have encouraged their participation in vaccination efforts, irrespective of their levels of C&CHL. Evidence from prior outbreaks, such as Ebola and SARS, has demonstrated that high trust in authorities led to greater adherence to preventative regulations [26, 27]. This could explain the high proportion of older people adhering to the recommendation of getting vaccinated against COVID-19, as most people in Sweden trust healthcare authorities [28].

### Refraining from health or dental care

Our study shows that 10% of participants refrained from seeking health or dental care when needed due to fear of COVID-19 infection. Although this is a relatively small number, the implications of not seeking care when needed can be profound, especially among the older population [29]. This should be further studied to understand what could be done to prevent people from accessing necessary care. However, refraining from health or dental care was not associated with C&CHL. This contrasts with most studies on the association between fear of COVID-19 infection and HL, which have shown that higher HL is associated with a lower fear of COVID-19 and a lower level of refraining from seeking health care [30], regardless of their fear of COVID-19 infection.

### Methodological considerations

The main strength of this study is minimising the knowledge gap. To our knowledge, this is the first population-based study in Sweden evaluating the link between C&CHL levels and adherence to COVID-19 recommendations.

Although the study is cross-sectional and therefore prevents drawing any conclusions about causality, many of the independent variables used in the regression analysis are relatively stable or immutable in this older population (e.g., sex, educational level, social class based on previous occupation). They were chosen based on results from prior research [4, 25, 29].

Data were collected over a 12-month period (between May 2021 and April 2022) during which public health restrictions evolved, which may have influenced how participants perceived and reported their adherence; however, the core recommendations remained largely consistent throughout. The data collection period also coincided with the peak of the national vaccination campaign. The vaccination programme was implemented in four sequential priority stages. The first stage included older people in assisted living residential care and health or elderly care workers. Again, stage three included individuals aged 60–64 and various risk groups aged 18–59 by April 26, 2021 [1]. This means that, at the time of data collection, the study population had received at least one dose of the COVID-19 vaccination. This reflects Sweden’s vaccination initiatives, which ensured a timely and representative overview of vaccine uptake among priority groups, minimising recall bias and providing insights consistent with active public health recommendations.

One study limitation is that we could not include older people with severe cognitive impairment (24%) as the dataset lacked the information required to assess their level of C&CHL. However, these individuals may have limited C&CHL, including cognitive abilities, such as accessing, understanding, appraising, and using health information. This exclusion may have resulted in the overestimation of the overall C&CHL levels in the older population of this study, leading to an underrepresentation of the challenges faced by this vulnerable group and potentially impacting the representativeness of the findings for the broader older population in Sweden.

Another limitation is the crude measure of education used in this study. We used this categorisation because the education categories changed between cohorts, making it difficult to distinguish between different levels of education in the study. Also, the sample includes a slightly higher proportion of individuals with higher education levels, which may introduce some bias and limit the generalisability of the findings to subgroups with lower educational attainment or health literacy.

Furthermore, since the survey was conducted in the Swedish language, participants with immigrant backgrounds were excluded, despite their high prevalence of COVID-19 infections and severe outcomes globally [31] and in Sweden compared to natives [32]. This may have resulted in an underestimation of the actual number of people who responded to the health recommendations associated with the C&CHL in our study. Specific studies on the C&CHL of older migrant people and how they respond to COVID-19 health recommendations would be valuable.

While HL is an individual-level skill, addressing it requires a societal-level approach by improving organisational HL (OHL). Although OHL is not directly measured in this study, it provides essential context: the finding that most older individuals adhered to recommendations regardless of HL level may indicate that these public health messages were successfully delivered in an HL-responsive manner. In other words, our results may indirectly reflect the impact of effective OHL in practice.

## Conclusions

Swedish COVID-19 recommendations have been disseminated in an HL-responsive way. Regardless of individual C&CHL level, most older people understood and followed the recommendations, and almost everyone received a vaccination. However, among the few who needed help understanding the recommendations, a higher proportion had limited C&CHL. Subgroup analyses on older migrant populations were not possible, and further studies are required to determine whether these results are also generalisable to this group.
